# Cocaine-induced midline destruction

**DOI:** 10.1093/omcr/omaa135

**Published:** 2021-02-15

**Authors:** Veerle Ide, Liesbet Henckaerts, Peter Vanbrabant, Steven Vanderschueren

**Affiliations:** Clinical Department of General Internal Medicine, University Hospitals Leuven, Research Department of Microbiology, Immunology and Transplantation, Laboratory for Clinical Infectious and Inflammatory Disorders, KU Leuven, Herestraat 49, Leuven, Belgium

**Keywords:** Emergency medicine, Radiology, Nuclear medicine and medical imaging, Rheumatology, Substance abuse, Audiovestibular Medicine

## Abstract

In patients presenting with nasal septum perforation, the differential diagnosis between ANCA-associated vasculitis and cocaine-induced midline destruction (CIMD) can be challenging. We describe the case of a 28-year old man who presented with a nasal septum perforation. He admitted the use of cocaine and showed no other symptoms of systemic inflammation. Perinuclear anti-neutrophilic cytoplasmatic antibodies (p-ANCAs) came back positive, as did anti-proteinase 3-antibodies. Further testing revealed antibodies to human neutrophil elastase (HNE), typically found in CIMD but rarely in ANCA-associated vasculitis. The combination of an atypical ANCA-pattern and the detection of HNE-antibodies led to the diagnosis of CIMD. In conclusion, HNE antibodies can be used to distinguish between CIMD and ANCA-associated vasculitis.

## MANUSCRIPT

**Figure 1:**
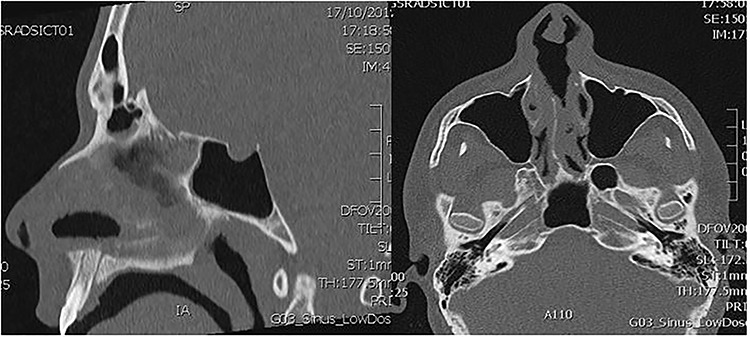
Sagittal and transverse computed tomography section through the nasal septum showing a nasal septum perforation.

A 28-year old man was admitted to the hospital with complaints of pain in the nose, nasal obstruction, rhinorrhea and headache since 3 months. He already received a 3-week course of antibiotics (Amoxicillin–clavulanate) without improvement. Laboratory analysis demonstrated inflammation with a C-reactive protein of 77 mg/L and mild leukocytosis (11.7 × 10^9^/L) with eosinophilia. Rhinoscopy showed a large nasal septum perforation. Nasal endoscopy revealed extensive necrosis and scabs. A computed tomography sinuses confirmed the presence of a nasal septum perforation (Fig. 1). When asked about it, the patient admitted the use of cocaine, and also the urinary toxicology test was strongly positive for this drug. The diagnosis of a cocaine-induced midline destruction (CIMD) was made.

As a nasal septum perforation can also be the presenting symptom of an ANCA-associated vasculitis, such as (limited) granulomatosis with polyangiitis (GPA), anti-neutrophilic cytoplasmatic antibodies (ANCAs) were measured. Perinuclear ANCAs (p-ANCAs) came back positive (titer 1/80), as did anti-proteinase 3 (anti-PR3) antibodies (9.7 U/mL, considered positive if >3 U/mL). Myeloperoxidase antibodies were negative. Further testing revealed antibodies to human neutrophil elastase (HNE).

Literature data show that patients with CIMD often have positive ANCAs, which makes it difficult to differentiate between CIMD and ANCA-associated vasculitis [1, 2]. However, positive ANCAs in CIMD are often seen in atypical patterns, for example p-ANCAs in combination with anti-PR3-antibodies (whereas with GPA, one would expect c-ANCAs directed against PR3). Furthermore, ANCAs in CIMD are often targeted against atypical antigens such as HNE whereas HNE-antibodies are rarely found in patients with ANCA-associated vasculitis [2–4]. HNE antibodies can thus be used to distinguish between ANCA-associated vasculitis and CIMD, preventing the unnecessary use of immunosuppressive agents [2, 4]. Treatment of CIMD focuses mainly on symptom relief. Stop of cocaine use is of primary importance to prevent further damage [4]. Psychological counseling is recommended given the addictive features of cocaine.

## CONFLICT OF INTEREST STATEMENT

There is no conflict of interest.

## FUNDING

There was no funding for this publication.

## ETHICAL APPROVAL

No ethical approval required.

## CONSENT

Informed consent was obtained from the patient.

## GUARANTOR

Dr Veerle Ide is the guarantor for this publication.

## References

[1] Mirzaei A, Zabihiyeganeh M, Haqiqi A Differentiation of cocaine-induced midline destructive lesions from ANCA-associated Vasculitis. Iran J Otorhinolaryngol 2018;30:309–13. 30245987 PMC6147272

[2] Wiesner O, Russell KA, Lee AS, Jenne DE, Trimarchi M, Gregorini G, et al. Antineutrophil cytoplasmic antibodies reacting with human neutrophil elastase as a diagnostic marker for cocaine-induced midline destructive lesions but not autoimmune vasculitis. Arthritis Rheum 2004;50:2954–65. 15457464 10.1002/art.20479

[3] Trimarchi M, Bussi M, Sinico RA, Meroni P, Specks U Cocaine-induced midline destructive lesions - an autoimmune disease? Autoimmun Rev 2013;12:496–500. 22940554 10.1016/j.autrev.2012.08.009

[4] Graf J Rheumatic manifestations of cocaine use. Curr Opin Rheumatol 2013;25:50–5. 23196324 10.1097/BOR.0b013e32835b4449

